# Supplementary material for: Durotaxis is a driver and potential therapeutic target in lung fibrosis and metastatic pancreatic cancer
Source: Nat Cell Biol. 2025 Sep 9;27(9):1543–54. doi: 10.1038/s41556-025-01697-8 (PMC12431851; doi:10.1038/s41556-025-01697-8)
Supplement: Supplementary file 1 — Supplementary Methods. [file 41556_2025_1697_MOESM1_ESM.pdf]

nature cell biology

Supplementary information https://doi.org/10.1038/s41556-025-01697-8

Durotaxis is a driver and potential therapeutic target in lung fibrosis and metastatic pancreatic cancer

In the format provided by the authors and unedited

Durotaxis is a driver and therapeutic target in organ fibrosis and metastatic pancreatic cancer

Taslim A. Al-Hilal#[1,2,3,4], Maria-Anna Chrysovergi#[1,2,3], Paula E. Grasberger[1,2,3], Fei Liu[5], Vera Auernheimer[6], Yan Zhou[1,2,3], Zebin Xiao[7], Mark Anthony Leon Duque[8], Alba Santos[1,2,3], Tamanna Islam[4], Matteo Ligorio[9], Delphine Sicard[10], Clemens K. Probst[1,2,3], Vladimir Vrbanac[3], Tejaswini S. Reddi[11], Ludovic Vincent[12,13], Cassandra Happe[12,13], Edward Chaum[14], Charles R. Yates[15], Kaveh Daneshvar[16], Allan C. Mullen[16], David Ting[9], Eric S. White[17], Raghu Kalluri[18], Christina M. Woo[8], Ellen Puré[7], Wolfgang H. Goldmann[6], Jose Luis Alonso[19], Andrew M. Tager[1,2,3]†, Adam J. Engler[12,13], Daniel J. Tschumperlin[10] and David Lagares*[1,2,3,20]

Supplementary Methods

Human primary healthy and IPF fibroblasts

Healthy lung fibroblasts were isolated from lung sections from patients without IPF that underwent lung transplant. Cells were cultured in Dulbecco's modified Eagle medium (DMEM, Lonza) supplemented with 10% FBS (Lonza), 2 mM L-glutamine (Lonza), 1% penicillin-streptomycin (Lonza) in a humidified incubator with 5% CO_2 at 37°C. For culture maintenance, media was changed every three days and cells passaged before reaching confluence. Cells between passages 3–8 were used for all experiments. All lines were tested for mycoplasma contaminations.

Patient-Derived PDAC and CAF cell lines

PDAC 2, PDAC 3, PDAC 9, PDAC 5, PDAC6 and PDAC8 tumor cell lines and primary cancer associated fibroblast (CAF) line were kindly provided by Drs. David Ting and Matteo Ligorio (MGH Cancer Center and Harvard Medical School, Boston, USA). These patient-derived PDAC cell lines were derived from metastatic ascites from patients under a discarded tissue protocol in accordance with the Massachusetts General Hospital (MGH) IRB protocol 2011P001236. Tumor cell lines were immortalized, constitutively expressing a GFP-Luciferase construct ([74]. CAFs were similarly immortalized for continual culturing by infecting with hTERT (pBABE-hygro-hTERT), constitutively expressing mCherry. Cells were cultured in DMEM with 10% FBS (Lonza), 2 mM L-glutamine (Lonza) and 1% penicillin-streptomycin (Lonza). All lines were tested for mycoplasma contaminations.

KPC689 mouse pancreatic tumor line

The KPC689 cancer cell line was kindly provided by Dr. Raghu Kalluri (MD Anderson Cancer Center, Houston, Texas, USA) and established from the pancreatic tumors of Pdx1cre/+;LSL-KRasG12D/+;LSL-Trp53R172H/+ (KPC) mice, as previously described [77,78]. KPC689 cells were engineered to stably express GFP and luciferase following infection with F-Luc-GFP lentivirus (Capital Biosciences) and cultured in α-MEM supplemented with 1% L-glutamine and 1% penicillin-streptomycin. All lines were tested for mycoplasma contaminations.

Human and mouse cell lines

Mesenchymal stem cells (MSCs, Lonza), human lung fibroblasts (IMR-90, ATCC, CCL-186), human foreskin fibroblasts (ATCC, SCRC-1041), primary normal dermal fibroblasts (ATCC, PCS-201-010), human primary kidney fibroblasts (Cell Biologics, H-6016), human primary liver fibroblasts (ATCC, FL 62891), primary umbilical vein endothelial cells (HUVEC, ATCC, PCS-100-013), human embryonic kidney cells (293T, ATCC, CRL-1573) were purchased from commercial vendors. Primary lung mouse fibroblasts, mouse leukocytes, and mouse lung endothelial cells (MLEC) were isolated from C57Bl6 mice by tissue-digestion process, as previously described [54,55].

Antibodies, reagents, lentiviruses

Antibodies used were as follows: α-SMA (1A4, Sigma-Aldrich); Phospho-FAK (Tyr397) (#3283, Cell Signaling), FAK (#3285, Cell Signaling), Phospho-Paxillin (Tyr118) (MAB61641, R&D), Phospho-Paxillin (Tyr31) (#2541, Cell Signaling), Paxillin (MA124952, Invitrogen), Chicken Paxillin (Clone: PXC-10, Invitrogen), GFP (#2555, Cell Signaling), YAP (H-9, sc-271134, Santa

Cruz Biotechnologies), CD31 (PECAM-1) (#77699, Cell Signaling), STAT3 (12640, Cell Signaling), Phospho-Stat3 (Tyr705) (D3A7) (9145, Cell Signaling) laminin-β1 (LT3, sc-33709, Santa Cruz), laminin-332 (711306, Invitrogen), β-actin (#4970, Cell Signaling), GAPDH (glyceraldehyde-3-phosphate dehydrogenase) (Cell Signaling). Secondary antibodies were obtained from Invitrogen [Alexa Fluor 488 goat anti-mouse immunoglobulin G2a (IgG2a) and Alexa Fluor 555 goat anti-rabbit IgG1]. F-actin and nuclei were stained with Alexa Fluor 546–phalloidin and 4′,6-diamidino-2-phenylindole (DAPI) (Invitrogen), respectively. Antibody dilutions were prepared according to the manufacturer's guidelines

Mouse and human recombinant TGF-β1 and PDGF-BB were purchased from R&D Systems, and 18:1 LPA was purchased from Avanti Polar Lipids. Fibronectin and collagen type I were obtained from Sigma-Aldrich.

Reagents used included pirfenidone, nintedanib, BrdU (5-Bromo-2′-deoxyuridine), tamoxifen, genipin and glycerol from Sigma-Aldrich. JP-153 was provided by Dr. Charles Y. Ryan (The University of Mississippi School of Pharmacy, USA).

Chemotaxis assay

Chemotaxis was assayed using a 96-Multiwell FluoroBlok Inserting system (Fisher Scientific; pore size: 8 μm) precoated with fibronectin at 10 μg/ml (Sigma). Briefly, cells were labeled with DiIC12(3) fluorescent dye for 1 h before chemotaxis. 50,000 cells in 50 μl serum-free DMEM were then added to the apical chambers and exposed to 18:1 LPA (Avanti Polar Lipids) or PDGF-BB (R&D Systems) as chemoattractants. The plate was then incubated for 4 h at 37˚C in a 5% CO_2 atmosphere. Fluorescence of migrated cells was recorded at 544/590 nm (Ex/Em) on a bottom-reading fluorescent plate reader using Fluoroskan Ascent FL (Thermo). The data were expressed as the ratio of migrated cells toward any chemoattractant to cells migrated toward serum-free DMEM. Experiments were performed in triplicate.

Haptotaxis assay

Haptotaxis was assayed using μ-Slide microfluidic chambers (Ibidi) coated with 50 μg/ml fibronectin or 100 μg/m collagen type I solution. Briefly, 14 μl of fibronectin or collagen type I were loaded through port 6. The chamber was incubated for 1 hour at room temperature; unbound fibronectin was then washed out with PBS. Cells in 6 μl (4×10^5 cells/ml) were loaded into the central channel of the chamber and cells tracked for 24 hours.The haptotactic index and the FMI were evaluated using the ibidi Migration Tool, according to the manufacturer's instructions. Experiments were performed in triplicate.

Invasion assay

Invasion was assayed using a 96-well BioCoat Tumor Invasion System (Fisher Scientific). 50,000 cells in 50 μl serum-free DMEM were then added to the apical chambers and exposed to 10% FBS DMEM media as chemoattractant. The plate was read at 48 h and the data expressed as the ratio of invaded cells toward any chemoattractant to cells migrated toward serum-free DMEM. Experiments were performed in triplicate.

Proliferation assay

Cells were seeded in 96 well plates at 5,000 cells/well in DMEM supplemented with 0% or 10% FBS and incubated for 18–24 h at 37˚C to allow for adhesion. Cells were then treated with

inhibitors and incubated for 72 h at 37˚C, 5% CO2. CCK-8 solution (10 µL) was added to each well and allowed to incubate for 1 h at 37 ˚C, 5% CO2 and absorbance was measured at 450 nm. Experiments were performed in triplicate.

Viability assay

Cells were seeded in 96 well plates at 5,000 cells/well in DMEM supplemented with 0% or 10% FBS and incubated for 18–24 h at 37˚C to allow for adhesion. Cells were then treated with inhibitors in serum-free medium for 96 h. Cells were washed twice with PBS and 50 µL of 0.5% crystal violet staining solution was added to each well and incubated for 20 min at RT. Next, each well was washed with PBS four times and allowed to air dry for 4 h at 25 ˚C. Methanol (200 µL) was added to each well and the plate was incubated for 20 min at 25 ˚C on a rocker. Absorbance was then measured at 570 nm. Experiments were performed in triplicate.

siRNA and plasmid transfection

The following expression constructs were provided by Dr. Clare M. Waterman (NHLBI): wild type human paxillin (paxillin^{WT}), constitutively active human paxillin phosphomimetic (paxillin$^{Y31/118E}$), inactive non-phosphorylatable human paxillin (paxillin$^{Y31/118F}$), human paxillin point mutant defective in vinculin binding (Paxillin^{E151Q}), and wild type chicken paxillin. Transient transfection experiments with paxillin mutants were performed on primary human and mouse lung fibroblasts seeded on 6-well plates (60–70% confluency) using Lipofectamine 2000 (Thermo Fisher Scientific).

The siRNA duplexes targeting human paxillin mRNA were On-Target Plus Smart Pools and were obtained from Dharmacon Inc. (Thermo Scientific). The siRNAs (20 nM) were transiently transfected into human and mouse primary fibroblasts using HiPerFect Reagent (Qiagen) at a siRNA/HiPerFect ratio of 1:4 (µg/µl). siRNAs from the On-Target Plus nontargeting siRNA pool were used as a nonspecific control. Cells were harvested and mRNA levels were assessed 48 h after transfection.

RT-PCR and qRT-PCR

Total RNA was extracted using Qiagen extraction kits according to the manufacturer's protocol, and cDNAs were generated by reverse transcription using iScript cDNA Synthesis Kit (BioRad). qPCR was performed using fluorogenic SYBR Green and Mx4000 Multiplex Quantitative PCR System (Stratagene). GAPDH was used as reference gene in all qRT-PCR reactions. PCR was performed using the primers listed in Supplementary Table 2 at a final concentration of 100 nM. Relative transcript abundance of a gene is expressed in ΔCt values (ΔCt = Ctreference – Cttarget). Relative changes in transcript levels compared with controls are expressed as ΔΔCt values (ΔΔCt = ΔCttreated – ΔCtcontrol).

Western blot analysis

Cells and tissues were harvested, lysed in RIPA buffer (Thermo Scientific) and supplemented with Halt Protease and Phosphatase Inhibitor Cocktail (Thermo Scientific). Protein extracts were subjected to centrifugation (6,000g) at 4 °C, and protein concentrations were determined using BCA assay (Pierce). Protein was separated on either NativePAGE Bis-Tris Gel System or 4–12% SDS-Tris-Glycine Protein Gel. Separated proteins were transferred onto polyvinylidene difluoride (PDVF) membranes (Invitrogen), and membranes were blocked with 5% nonfat dry milk in TBS and incubated with the indicated primary antibodies. After washing, membranes were incubated

with appropriate secondary, HRP-linked antibodies (Pierce). Proteins were visualized by enhanced chemiluminescence and autoradiography (ECL; Amersham Biosciences, GE Healthcare). Densitometry of the immunoblots was performed using ImageJ software (NIH).

Immunofluorescence

Cells were plated on glass coverslips (50 cells per mm2), treated with JP-153 or TGF-β as described and fixed with 4% PFA for 10–15 min. The cells were then washed three times with PBS, blocked and permeabilized for one hour in blocking solution (10% goat serum; 0.1% triton-X in PBS) and incubated overnight at 4 °C with a primary antibody against α-SMA, Paxillin, P-Paxillin, YAP (H-9, sc-271134, Santa Cruz Biotechnologies). The next day, samples were washed three times with PBS and incubated for one hour with anti-mouse or rabbit Alexa Fluor 488- or 546-conjugated antibody (Life Technologies) diluted 1:500 in PBS with 1% BSA, and rhodamine phalloidin antibodies (Thermo Fisher Scientific) diluted to 1:200 in blocking solution. Cells were washed three times with PBS mounted with VECTASHIELD Antifade Mounting Medium with DAPI (Vectorlabs). Images were acquired with a Zeiss LSM780 confocal microscope (Zeiss). Focal adhesion size was analyzed using software MetaMorph 6.1.

Supplemental Methods: Generation of FAK^{L994E} knock-in (KI) mouse

(a)

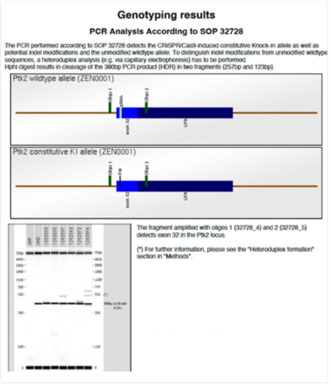

(b)

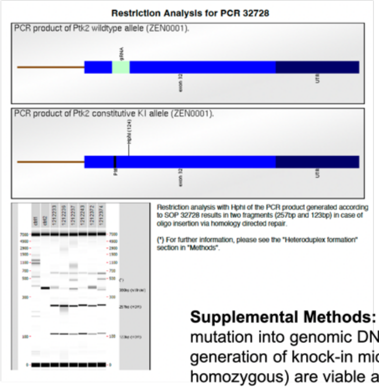

(c)

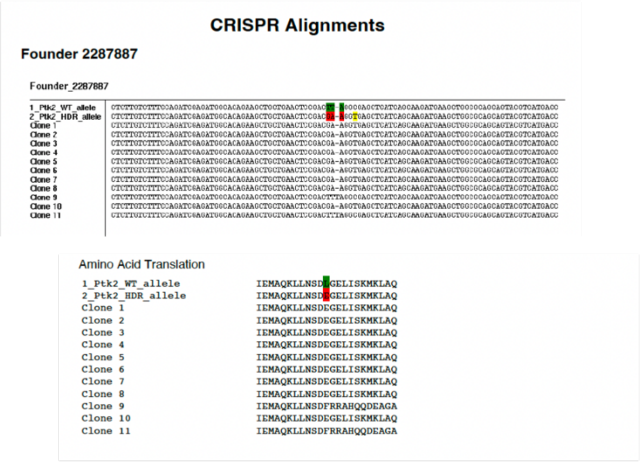

(d)

HC Female Inventory as of 04/29/2024				
Age in weeks	Date of Birth	Animal Line	Genotype	Quantity
4	04/01/2024	21473	(HET)	4
4	04/01/2024	21473	(HOM)	1
4	04/01/2024	21473	(WT)	1
6	03/18/2024	21473	(HET)	3
6	03/18/2024	21473	(HOM)	2
				11 Total

Supplemental Methods: (a, b) PCR-based genotyping and restriction analysis of PCR products confirming integration of the L994E mutation into genomic DNA. (c) DNA sequencing and CRISPR alignments studies confirm the integration of L994E point mutation and the generation of knock-in mice. Representative genotype of founder knock-in mouse is shown. (d) L994E knock-in mice (both het and homozygous) are viable and breed normally. Representative genotyping of female inventory as of 04/29/2024.
