# Supplementary material for: Durotaxis is a driver and potential therapeutic target in lung fibrosis and metastatic pancreatic cancer
Source: Nat Cell Biol. 2025 Sep 9;27(9):1543–54. doi: 10.1038/s41556-025-01697-8 (PMC12431851; doi:10.1038/s41556-025-01697-8)
Supplement: Supplementary file 2 — Reporting Summary [file 41556_2025_1697_MOESM2_ESM.pdf]

nature portfolio

Corresponding author(s): David Lagares

Last updated by author(s): Apr 24, 2025

Reporting Summary

Nature Portfolio wishes to improve the reproducibility of the work that we publish. This form provides structure for consistency and transparency in reporting. For further information on Nature Portfolio policies, see our Editorial Policies and the Editorial Policy Checklist.

Statistics

For all statistical analyses, confirm that the following items are present in the figure legend, table legend, main text, or Methods section.

n/a	Confirmed	
☐	☒	The exact sample size (*n*) for each experimental group/condition, given as a discrete number and unit of measurement
☐	☒	A statement on whether measurements were taken from distinct samples or whether the same sample was measured repeatedly
☐	☒	The statistical test(s) used AND whether they are one- or two-sided<br>*Only common tests should be described solely by name; describe more complex techniques in the Methods section.*
☐	☒	A description of all covariates tested
☐	☒	A description of any assumptions or corrections, such as tests of normality and adjustment for multiple comparisons
☐	☒	A full description of the statistical parameters including central tendency (e.g. means) or other basic estimates (e.g. regression coefficient) AND variation (e.g. standard deviation) or associated estimates of uncertainty (e.g. confidence intervals)
☐	☒	For null hypothesis testing, the test statistic (e.g. *F*, *t*, *r*) with confidence intervals, effect sizes, degrees of freedom and *P* value noted<br>*Give P values as exact values whenever suitable.*
☒	☐	For Bayesian analysis, information on the choice of priors and Markov chain Monte Carlo settings
☒	☐	For hierarchical and complex designs, identification of the appropriate level for tests and full reporting of outcomes
☒	☐	Estimates of effect sizes (e.g. Cohen's *d*, Pearson's *r*), indicating how they were calculated

Our web collection on statistics for biologists contains articles on many of the points above.

Software and code

Policy information about availability of computer code

Data collection	Only commercial software, included with the instruments described in the methods, was used in data collection:<br>AFM data was acquired using Bio-Catalyst AFM and MFP-3D AFM.<br>Imaging of the fresh mouse tumor slices was conducted using a Leica SP8-MP upright multiphoton microscope with a Coherent Chameleon Vision II MP laser, equipped.<br>Images were acquired with a Zeiss LSM780 confocal microscope (Zeiss).<br>Flow cytometry was performed using a BDLSR Fortessa X-20 cell analyzer.<br>IVIS imaging was use to visualize tumors in the pancreas, skin, livers and lungs.
Data analysis	Data was analyzed and visualized using Microsoft Excel and GraphPad Prism 5.0. DNA and protein sequences were analyzed using Geneious. Flow cytometry data was analyzed using FloJo. Images and time lapse videos were made and/or analyzed using FIJI, NIH ImageJ, MetaMorph 6.1, Manual Tracking Plugin and Correct 3D Drift Plugin. Two Photon Imaging Videos were generated by Imaris and LAS X software. Collagen measurement was conducted using CT-FIRE software. AFM data was analyzed using Bio-Catalyst AFM and MFP-3D AFM integrated softwares, resulting young modulus data was plotted in 3D stiffness maps using MATLAB.

For manuscripts utilizing custom algorithms or software that are central to the research but not yet described in published literature, software must be made available to editors and reviewers. We strongly encourage code deposition in a community repository (e.g. GitHub). See the Nature Portfolio guidelines for submitting code & software for further information.

Data

Policy information about availability of data

All manuscripts must include a data availability statement. This statement should provide the following information, where applicable:

- Accession codes, unique identifiers, or web links for publicly available datasets
- A description of any restrictions on data availability
- For clinical datasets or third party data, please ensure that the statement adheres to our policy

The authors declare that all data generated or analyzed during this study are available upon request. Requests for raw or additional data should be emailed to the corresponding author and should include a brief description of the proposed analysis. Requests for data access will be reviewed individually, and a decision will be communicated within 4weeks of receipt. Patient-derived data containing confidential or identifiable patient information are subject to patient privacy and cannot be shared. All data is available upon request.

Research involving human participants, their data, or biological material

Policy information about studies with human participants or human data. See also policy information about sex, gender (identity/presentation), and sexual orientation and race, ethnicity and racism.

Reporting on sex and gender	N/A
Reporting on race, ethnicity, or other socially relevant groupings	N/A
Population characteristics	N/A
Recruitment	N/A
Ethics oversight	All human experiments were performed under protocols approved by the Institutional Ethics Committee approved by the Massachusetts General Hospital. Patients with IPF were identified from those receiving care at the Massachusetts General Hospital. For study inclusion, patients with IPF had to satisfy IPF diagnostic criteria based on the 2011 joint consensus statement of the American Thoracic Society (ATS), European Respiratory Society (ERS), Japanese Respiratory Society, and Latin American Thoracic Association as determined by 2 investigators.

Note that full information on the approval of the study protocol must also be provided in the manuscript.

Field-specific reporting

Please select the one below that is the best fit for your research. If you are not sure, read the appropriate sections before making your selection.

☒ Life sciences ☐ Behavioural & social sciences ☐ Ecological, evolutionary & environmental sciences

For a reference copy of the document with all sections, see nature.com/documents/nr-reporting-summary-flat.pdf

Life sciences study design

All studies must disclose on these points even when the disclosure is negative.

Sample size

In each in vivo animal experiment evaluating the effects of genetic inhibition of the FAK-Paxillin pathway on the extent of dermal and lung fibrosis produced in mice, we use > or = 8 mice per group to achieve statistical significance and account for the inherent variability in the fibrotic response of mice. Assuming a 50% reduction in the amount of fibrosis present in FAK-Paxillin Knock-In mice compared with wild-type control mice, then at least 8 mice per group were needed to achieve a power of 80%, accepting a Type I error rate of 0.05. Histological analyses were done using n= 5 mice per group. Collagen determinations by hydroxyproline levels were performed using n=5 mice per group.

In each fibrosis experiment evaluating the effects of JP-153 inhibitor on the extent of lung fibrosis produced in mice, we use > or = 10 mice per group to achieve statistical significance and account for the inherent variability in the fibrotic response of mice. Assuming a 50% reduction in the amount of fibrosis present in mice treated with active drug compared with vehicle control, then 10 mice per group were needed to achieve a power of 80%, accepting a Type I error rate of 0.05. Collagen determinations by hydroxyproline levels were performed using n=6 mice per group, flow cytometry studies were performed using n= 4-6, histological analysis of lung tissues were done using n=6 mice per group. Western blot analyses from our in vivo mouse model of lung fibrosis includes 6 samples per condition. Representative blot shows at least n=3 samples/group.

In each tumor experiment evaluating the effects of genetic inhibition of FAK-Paxillin or pharmacological inhibition of the pathway with JP-153 inhibitor on tumor growth, tumor fibrosis and metastasis. Power analysis was used to determine the sample size of at least eight mice per group (80% power for an effect size of at least 1.5, assuming 5% significance level and a two-sided test).

Data exclusions

No data was excluded.

Replication	Number of replicates is described in the figure legends, where applicable. All attempts of replication were successful.
Randomization	For genetic or pharmacological experiments with JP-153 inhibitor, all mice used were wild type (C57Bl/6N) animals, and were purchased commercially and randomized to experimental groups by the cages our animal facility put them in upon their arrival from the vendor.
Blinding	The investigators were blinded to group allocation during data collection and analyses.

Reporting for specific materials, systems and methods

We require information from authors about some types of materials, experimental systems and methods used in many studies. Here, indicate whether each material, system or method listed is relevant to your study. If you are not sure if a list item applies to your research, read the appropriate section before selecting a response.

Materials & experimental systems

n/a	Involved in the study
☐	☒ Antibodies
☐	☒ Eukaryotic cell lines
☒	☐ Palaeontology and archaeology
☐	☒ Animals and other organisms
☒	☐ Clinical data
☒	☐ Dual use research of concern
☒	☐ Plants

Methods

n/a	Involved in the study
☒	☐ ChIP-seq
☐	☒ Flow cytometry
☒	☐ MRI-based neuroimaging

Antibodies

Antibodies used	Antibodies used were as follows: α-SMA (1A4, Sigma-Aldrich); Phospho-FAK (Tyr397) (#3283, Cell Signaling), FAK (#3285, Cell Signaling), Phospho-Paxillin (Tyr118) (MAB61641, R&D), Phospho-Paxillin (Tyr31) (#2541, Cell Signaling), Paxillin (MA124952, Invitrogen), Chicken Paxillin (Clone: PXC-10, Invitrogen), GFP (#2555, Cell Signaling), YAP (H-9, sc-271134, Santa Cruz Biotechnologies), CD31 (PECAM-1) (#77699, Cell Signaling), STAT3 (12640, Cell Signaling), Phospho-Stat3 (Tyr705) (D3A7) (9145, Cell Signaling) laminin-β1 (LT3, sc-33709, Santa Cruz), laminin-332 (711306, Invitrogen), β-actin (#4970, Cell Signaling), GAPDH (glyceraldehyde-3-phosphate dehydrogenase) (Cell Signaling). Secondary antibodies were obtained from Invitrogen [Alexa Fluor 488 goat anti-mouse immunoglobulin G2a (IgG2a) and Alexa Fluor 555 goat anti-rabbit IgG1]. F-actin and nuclei were stained with Alexa Fluor 546–phalloidin and 4′,6-diamidino-2-phenylindole (DAPI) (Invitrogen), respectively. Antibody dilutions were prepared according to the manufacturer's guidelines
Validation	Antibodies were used within the uses validated by the manufacturers.

Eukaryotic cell lines

Policy information about cell lines and Sex and Gender in Research

Cell line source(s)	Mesenchymal stem cells (MSCs, Lonza), human lung fibroblasts (IMR-90, ATCC, CCL-186), human foreskin fibroblasts (ATCC, SCRC-1041), primary normal dermal fibroblasts (ATCC, PCS-201-010), human primary kidney fibroblasts (Cell Biologics, H-6016), human primary liver fibroblasts (ATCC, FL 62891), primary umbilical vein endothelial cells (HUVEC, ATCC, PCS-100-013), human embryonic kidney cells (293T, ATCC, CRL-1573) were purchased from commercial vendors. Primary lung mouse fibroblasts, mouse leukocytes, and mouse lung endothelial cells (MLEC) were isolated from C57Bl6 mice by tissue-digestion process. Healthy lung fibroblasts were isolated from lung sections from patients without IPF that underwent lung transplant. PDAC 2, PDAC 3, PDAC 9, PDAC 5, PDAC6 and PDAC8 tumor cell lines and primary cancer associated fibroblast (CAF) line were kindly provided by Drs. David Ting and Matteo Ligorio (MGH Cancer Center and Harvard Medical School, Boston, USA). These patient-derived PDAC cell lines were derived from metastatic ascites from patients under a discarded tissue protocol in accordance with the Massachusetts General Hospital (MGH) IRB protocol 2011P001236. Tumor cell lines were immortalized, constitutively expressing a GFP-Luciferase construct (74. CAFs were similarly immortalized for continual culturing by infecting with hTERT (pBABE-hygro-hTERT). The KPC689 cancer cell line was kindly provided by Dr. Raghu Kalluri (MD Anderson Cancer Center, Houston, Texas, USA) and established from the pancreatic tumors of Pdx1cre/+;LSL-KRasG12D/+;LSL-Trp53R172H/+ (KPC) mice.
Authentication	Commercial lines were validated by the vendors. No authentication was performed on primary cell cultures beyond positive/negative selection methods.
Mycoplasma contamination	All cells used were tested negative for mycoplasma. Mycoplasma testing is performed quarterly.
Commonly misidentified lines (See ICLAC register)	No commonly misidentified lines were used.

Animals and other research organisms

Policy information about studies involving animals; ARRIVE guidelines recommended for reporting animal research, and Sex and Gender in Research

Laboratory animals	Pathogen-free male C57BL/6N (6- to 8-week-old) mice purchased from the National Cancer Institute (NCI) Frederick Mouse Repository were used for mouse models of skin, lung and kidney fibrosis as well syngeneic tumor models. Immunocompromised NOD/SCID/gamma-c (NSG; NOD.Cg-Prkdcscid Il2rgtm1Wjl/Sz, 6- to 8-week-old) obtained from Jackson Laboratories were housed used for the orthotopic xenograft model.
Wild animals	This study did not involve wild animals.
Reporting on sex	We used mainly male mice for fibrosis animal experiments because males have higher susceptibility to bleomycin injury and develop more severe fibrosis in both skin and lungs, compared with female mice. For tumor xenograft experiments, all mice were female and 4-6 weeks old.
Field-collected samples	*For laboratory work with field-collected samples, describe all relevant parameters such as housing, maintenance, temperature, photoperiod and end-of-experiment protocol OR state that the study did not involve samples collected from the field.*
Ethics oversight	All experiments were performed in accordance with National Institute of Health guidelines and protocols were approved by the Massachusetts General Hospital Subcommittee on Research Animal Care.All mice were maintained in a specific pathogen–free (SPF) environment certified by the American Association for Accreditation of Laboratory Animal Care (AAALAC).

Note that full information on the approval of the study protocol must also be provided in the manuscript.

Plants

Seed stocks	*Report on the source of all seed stocks or other plant material used. If applicable, state the seed stock centre and catalogue number. If plant specimens were collected from the field, describe the collection location, date and sampling procedures.*
Novel plant genotypes	*Describe the methods by which all novel plant genotypes were produced. This includes those generated by transgenic approaches, gene editing, chemical/radiation-based mutagenesis and hybridization. For transgenic lines, describe the transformation method, the number of independent lines analyzed and the generation upon which experiments were performed. For gene-edited lines, describe the editor used, the endogenous sequence targeted for editing, the targeting guide RNA sequence (if applicable) and how the editor was applied.*
Authentication	*Describe any authentication procedures for each seed stock used or novel genotype generated. Describe any experiments used to assess the effect of a mutation and, where applicable, how potential secondary effects (e.g. second site T-DNA insertions, mosiacism, off-target gene editing) were examined.*

Flow Cytometry

Plots

Confirm that:

☒ The axis labels state the marker and fluorochrome used (e.g. CD4-FITC).

☒ The axis scales are clearly visible. Include numbers along axes only for bottom left plot of group (a 'group' is an analysis of identical markers).

☒ All plots are contour plots with outliers or pseudocolor plots.

☒ A numerical value for number of cells or percentage (with statistics) is provided.

Methodology

Sample preparation	Single-cell suspensions were isolated from mouse lung tissues biopsies using Liberase Blendzyme (final concentration, 0.14U/ml; Roche) and deoxyribonuclease I (final concentration, 60 mg/ml; Sigma) for 45 min at 37°C. Cells were incubated with FcRII and FcRIII blocking antibody (BioLegend, clone 93) for 10 min at 4°C followed by staining with the following fluorophore-conjugated antibody from Biolegend: Viability eF780 (1:1000), CD11b-BUV737 (1:100), Ly6G-FITC (1:200), Ly6C-PerCP-Cy5.5 (1:200), CCR2-PE (1:50), CD11c-BV605 (1:200), MHCII-Pe-Cy7 (1:1000), F4/80-PE (1:100), MerTK-APC (1:100), CD3-BUV395 (1:200), CD4-BV786 (1:200), CD8-FITC (1:200).
Instrument	Flow cytometry was performed using a BDLSR Fortessa X-20 cell analyzer
Software	FlowJo software V10 was used for analysis.
Cell population abundance	At least 10.000 cells were analyzed for each sample

Gating strategy	Initial cell population gating (SSC vs FSC) was adopted to exclude the debris. A figure exemplifying the gating strategy is provide in the supplementary information.

☒ Tick this box to confirm that a figure exemplifying the gating strategy is provided in the Supplementary Information.
