# Supplementary material for: Durotaxis is a driver and potential therapeutic target in lung fibrosis and metastatic pancreatic cancer
Source: Nat Cell Biol. 2025 Sep 9;27(9):1543–54. doi: 10.1038/s41556-025-01697-8 (PMC12431851; doi:10.1038/s41556-025-01697-8)
Supplement: Supplementary file 6 — Unprocessed western blots and/or gels. [file 41556_2025_1697_MOESM6_ESM.pdf]

Uncropped scans of all blots

P-FAK

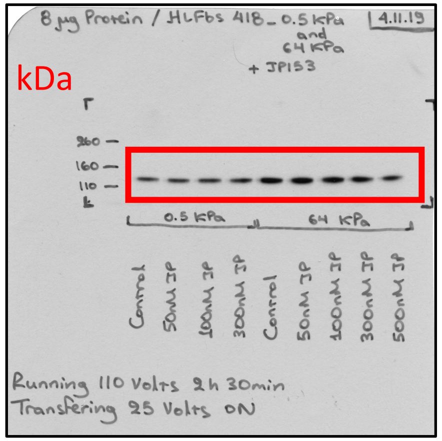

Fig. 3G

Total FAK

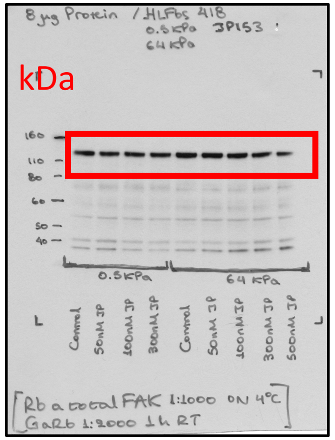

Fig. 3G

P-Paxillin

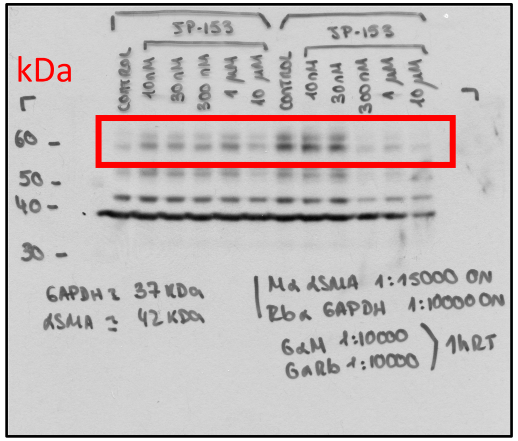

Fig. 3G

Total Paxillin

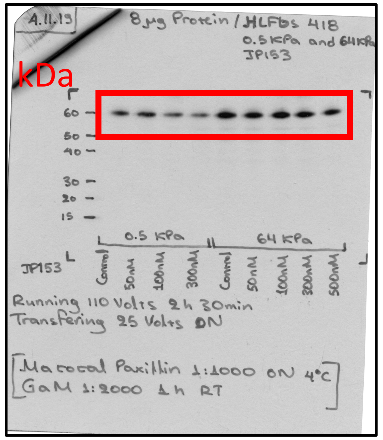

Fig. 3G

Beta Actin

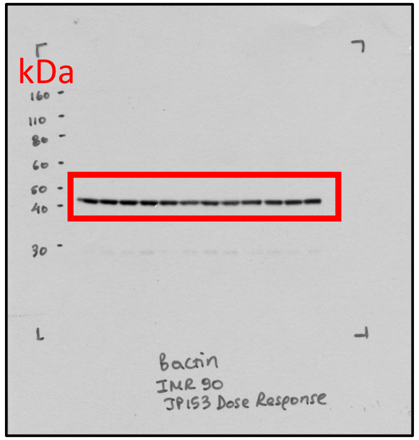

Fig. 3G

P-FAK

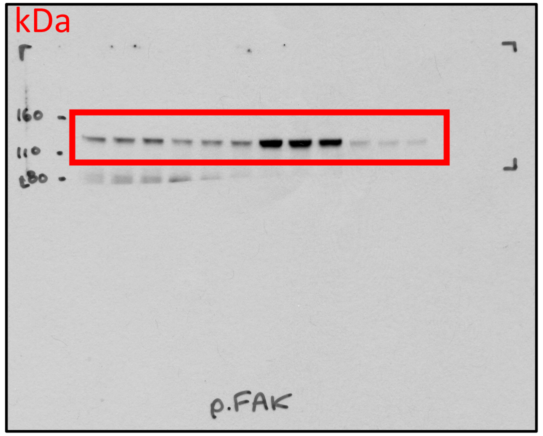

Fig. 4L

Total FAK

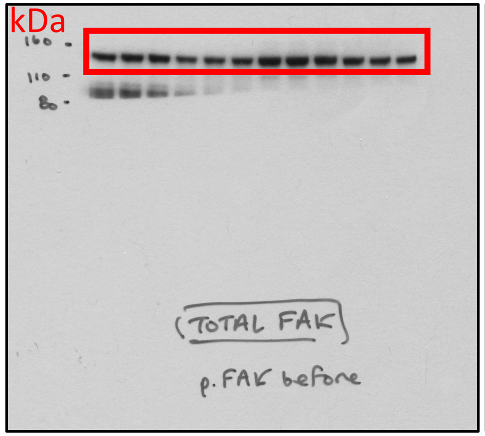

Fig. 4L

P-Paxillin

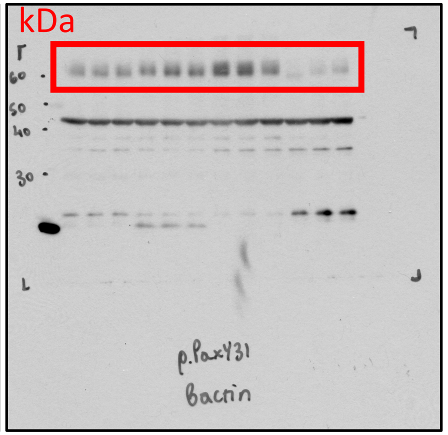

Fig. 4L

Total Paxillin

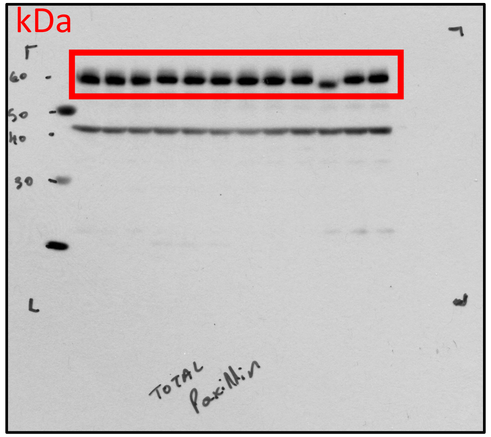

Fig. 4L

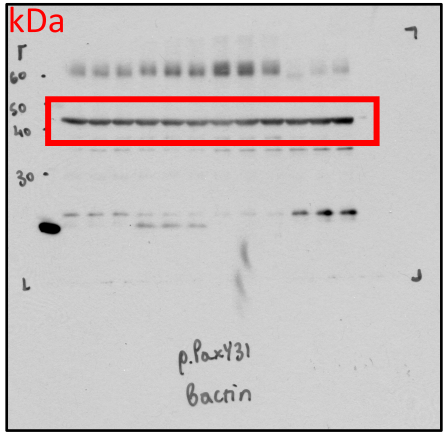

Fig. 4L

Uncropped scans of all blots

P-FAK

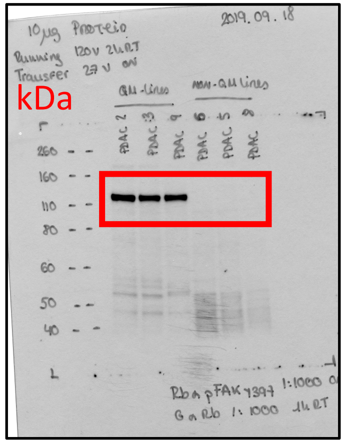

Fig. 5L

Total FAK

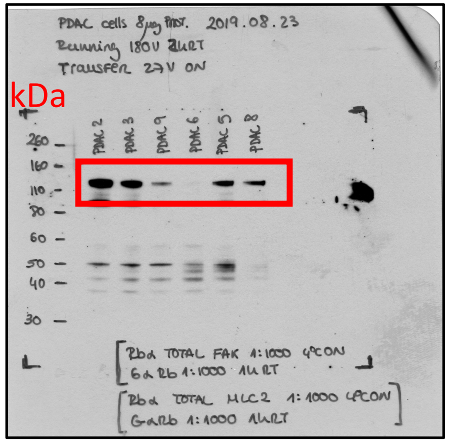

Fig. 5L

P-Paxillin

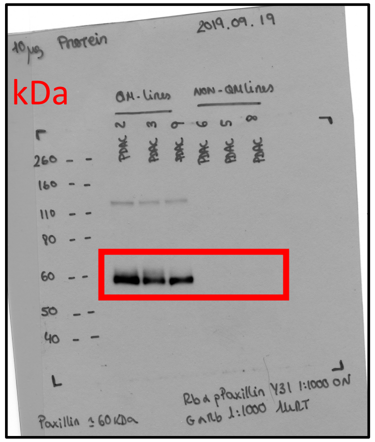

Fig. 5L

Total Paxillin

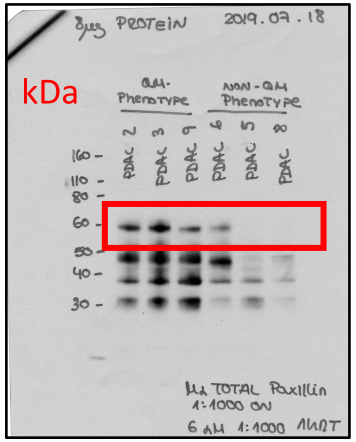

Fig. 5L

GAPDH

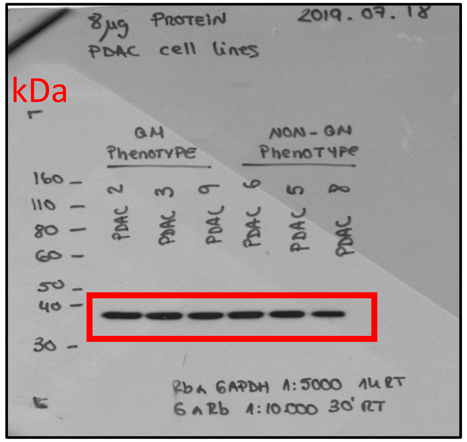

Fig. 5L

WT Paxillin

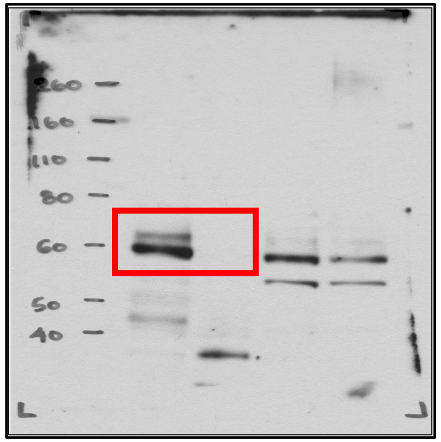

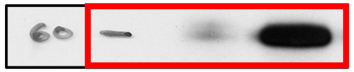

Mut Paxillin

GAPDH

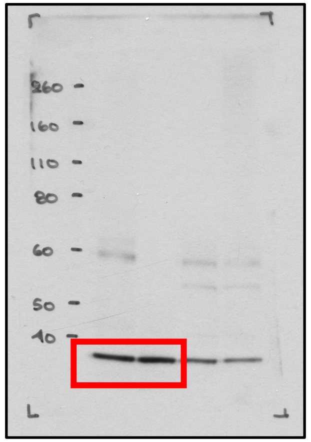

Fig. 5N
