# Supplementary material for: Durotaxis is a driver and potential therapeutic target in lung fibrosis and metastatic pancreatic cancer
Source: Nat Cell Biol. 2025 Sep 9;27(9):1543–54. doi: 10.1038/s41556-025-01697-8 (PMC12431851; doi:10.1038/s41556-025-01697-8)

Gating schemes for flow cytometry data shown in Fig. 4Q and Fig. 6S-U

Representative Gating strategy: Live/Epcam-/PDGFRa-/CD31-/CD45+

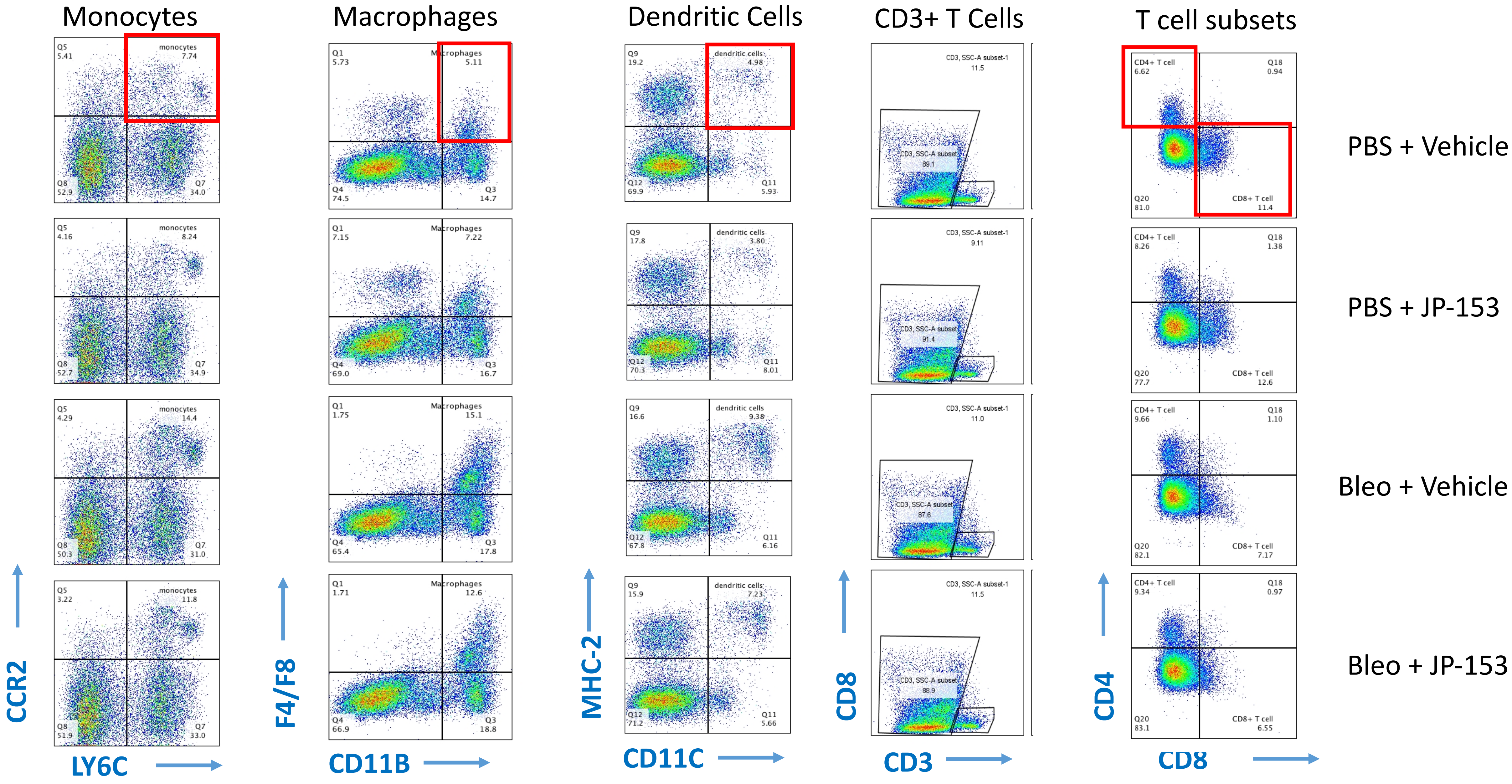

Supplement: Supplementary file 7 — Gating strategy for FACS. [file 41556_2025_1697_MOESM7_ESM.pdf]
